# Inclusion of enclosed hydration effects in the binding free energy estimation of dopamine D3 receptor complexes

**DOI:** 10.1371/journal.pone.0222902

**Published:** 2019-09-30

**Authors:** Rajat Kumar Pal, Satishkumar Gadhiya, Steven Ramsey, Pierpaolo Cordone, Lauren Wickstrom, Wayne W. Harding, Tom Kurtzman, Emilio Gallicchio

**Affiliations:** 1 Department of Chemistry, Brooklyn College, 2900 Bedford Avenue, Brooklyn, NY 11210, United States of America; 2 PhD Program in Biochemistry, The Graduate Center of the City University of New York, New York, NY 10016, United States of America; 3 PhD Program in Chemistry, The Graduate Center of the City University of New York, New York, NY 10016, United States of America; 4 Department of Chemistry, Hunter College, 695 Park Avenue, NY 10065, United States of America; 5 Department of Chemistry, Lehman College, 250 Bedford Park Blvd. West, Bronx, NY 10468, United States of America; 6 Department of Science, Borough of Manhattan Community College, 199 Chambers Street, New York, NY 10007, United States of America; Universidade Nova de Lisboa Instituto de Tecnologia Quimica e Biologica, PORTUGAL

## Abstract

Confined hydration and conformational flexibility are some of the challenges encountered for the rational design of selective antagonists of G-protein coupled receptors. We present a set of C3-substituted (-)-stepholidine derivatives as potent binders of the dopamine D3 receptor. The compounds are characterized biochemically, as well as by computer modeling using a novel molecular dynamics-based alchemical binding free energy approach which incorporates the effect of the displacement of enclosed water molecules from the binding site. The free energy of displacement of specific hydration sites is obtained using the Hydration Site Analysis method with explicit solvation. This work underscores the critical role of confined hydration and conformational reorganization in the molecular recognition mechanism of dopamine receptors and illustrates the potential of binding free energy models to represent these key phenomena.

## Introduction

One critical aspect of molecular recognition is the change in the hydration structure and hydration energetics induced by ligand binding. [1–5] Water molecules trapped, for example, in hydrophobic pockets within the binding site can be energetically disfavored as well as entropically frustrated relative to bulk water. Hence, displacements of these water molecules by the ligand can significantly enhance binding. [6–9] These effects are particularly important when comparing a series of ligands of interest which differ in the way they displace enclosed water molecules. The rational design of ligands using these principles can lead to improvements of binding potency and receptor selectivity. [10]

There have been significant efforts towards the development of methodologies to model the thermodynamic parameters and structural properties of water molecules at the protein surfaces. [11–16] Most of these methods employ an explicit representation of the solvent, which is considered the “gold standard” for modeling macromolecular complexes in part because of the capability of accurate representation of specific hydration environments. It is challenging, however, to access the time scales required to sample the changes in hydration states and capturing the effects of water expulsion from protein binding sites induced by ligand binding. [14, 17–19] We have shown that the influence of confined hydration can be also represented by a customized AGBNP2 [20] implicit solvent model trained on Hydration Site Analysis (HSA) [6, 8] data obtained with explicit solvation. [9] We take advantage of the first-shell hydration component of the AGBNP2 (Analytical Generalized Born Non Polar) model. In AGBNP2, hydration spheres placed on the solute surface represent short-range solute-solvent interactions, such as hydrogen bonding, not accurately described by a dielectric continuum representation. Similarly, we model the thermodynamics of hydration sites within the binding pocket using AGBNP2 first-shell hydration spheres.

The primary purpose of this work is to explore the applicability of our hybrid implicit solvent approach to protein-ligand systems. The dopamine D3 receptor is an important medicinal target in which the ligand recognition mechanism is heavily influenced by hydration effects. Due to conformational variability, the complexities of hydration and molecular interaction networks, and the lack of extensive structural information, it has been very challenging, using conventional drug design and modeling approaches, to design selective antagonists against the dopamine D3 family of receptors. We believe that molecular dynamics free energy approaches combined with accurate modeling of hydration could be helpful in the design of more effective and more specific antagonists. [21–24]

Dopamine D3 receptors, which are part of the G-protein coupled receptor superfamily, are increasingly important as drug targets for the treatment of a number of pathological conditions such as Parkinson’s disease, schizophrenia and drug abuse. [25–27] Dopamine receptors are classified under two families and five sub-types: the D1 family, comprising the D1R and D5R receptors which stimulate the production of cAMP, and the D2 family, comprising the D2R, D3R and D4R receptors which have inhibitory functions in cAMP production and downstream signaling. While both these receptor families have been targeted for the treatment of neurological disorders, it has been challenging to design specific antagonists within the D2 receptor subfamily. Most of the drugs tested act as dual D2/D3 antagonists. [28–31] D2 receptor antagonism has been associated with serious neurological side effects. [32, 33] D3 receptors, on the other hand, which also have high affinity towards dopamine were observed to significantly affect synaptic transmission and can be potential targets in the treatment of neurological disorders, especially related to drug addiction and craving responses. [29, 34, 35]

The mechanism of antagonism of D3 receptors has been intensely studied to gain an understanding of how to develop potent and selective antagonists. [22, 28, 30, 36, 37] The crystal structure of the D3 receptor in complex with eticlopride, [28] a dual D2/D3 antagonist, has been very helpful in understanding the intermolecular interactions in the orthosteric binding site (OBS) of the D3 receptor. It also revealed a secondary binding site (SBS) which is believed to be a critical molecular recognition site. A recent study has also suggested the existence of a cryptic pocket in the orthosteric binding site (OBS) of the dopamine D3 receptor. [36] These important discoveries have provided valuable information for the development of D3 selective ligands. [22, 23]

**Fig 1:**
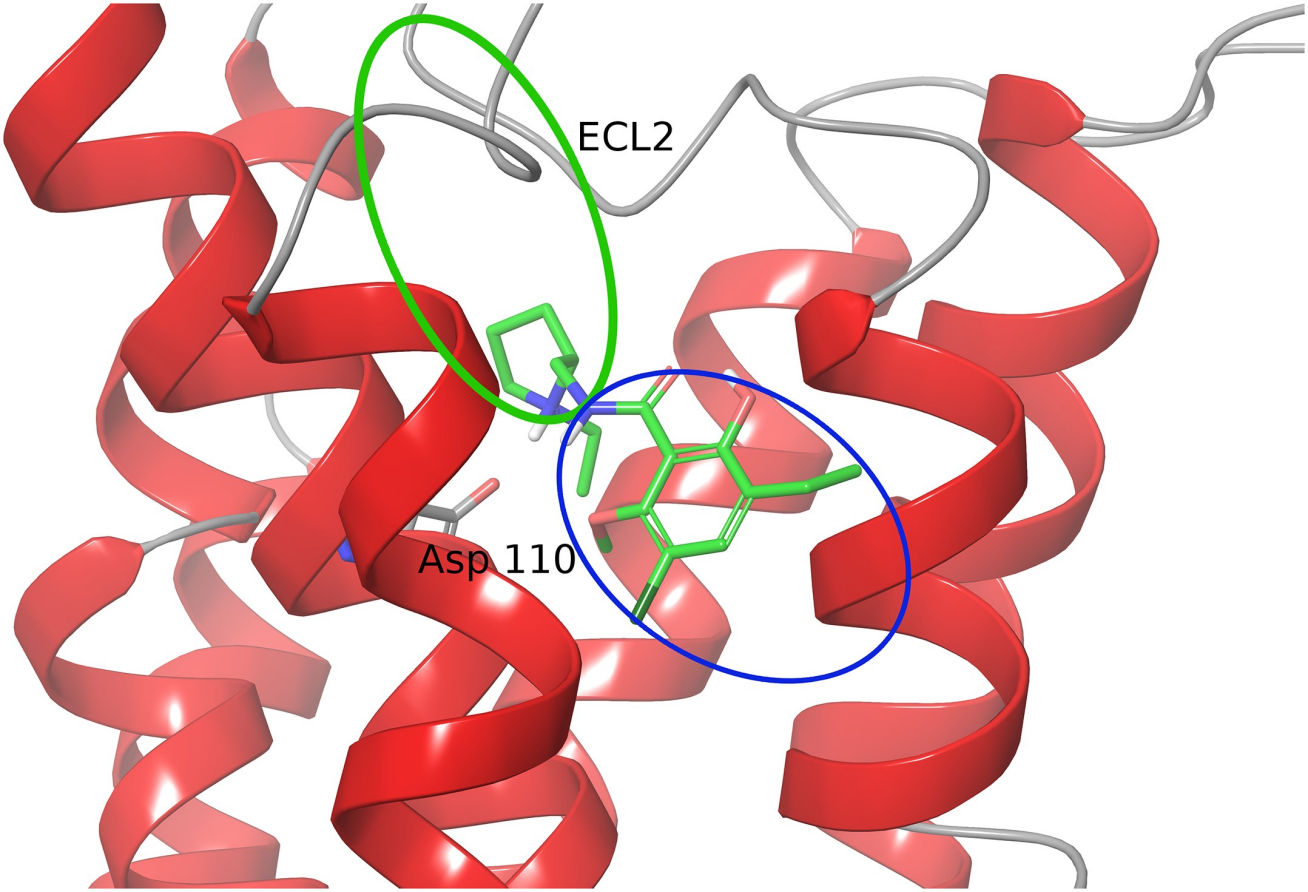
Crystal structure of the dopamine D3 receptor with eticlopride bound at the binding site. [28] This representation shows the approximate position of the orthosteric binding site (OBS) with a blue oval and the secondary binding site (SBS) with a green oval.

The orthosteric binding site (OBS) of D3 is surrounded by the helices III, V, VI and VII comprising Ser 192^5.42^, Ser 193^5.43^, Ser 196^5.46^, Cys 114^3.36^, His 349^6.55^, Phe 345^6.51^, Phe 346^6.52^ and Val 189^5.39^ residues. The secondary binding site (SBS), also referred as the extracellular extension, is located at the interface of helices I, II, III, VII and the extracellular loops ECL1 and ECL2 (Fig 1). The OBS is conserved in both D2 and D3 receptors but differ in the residue composition at the SBS. As exemplified by the structure of D3 bound to eticlopride [28] (Fig 1), the interaction of ligands to the OBS of D3 is characterized by a salt-bridge between the carboxylate group of Asp 110^3.32^ in helix III of D3 and the protonated amine group of eticlopride. This salt-bridge interaction is believed to be pharmacologically crucial in binding of ligands at the OBS of dopamine D3 receptor and to other dopaminergic receptors. [28] Previous studies have highlighted the challenges of designing specific antagonists against the dopamine D3 receptor. [21, 23, 37]

**Fig 2:**
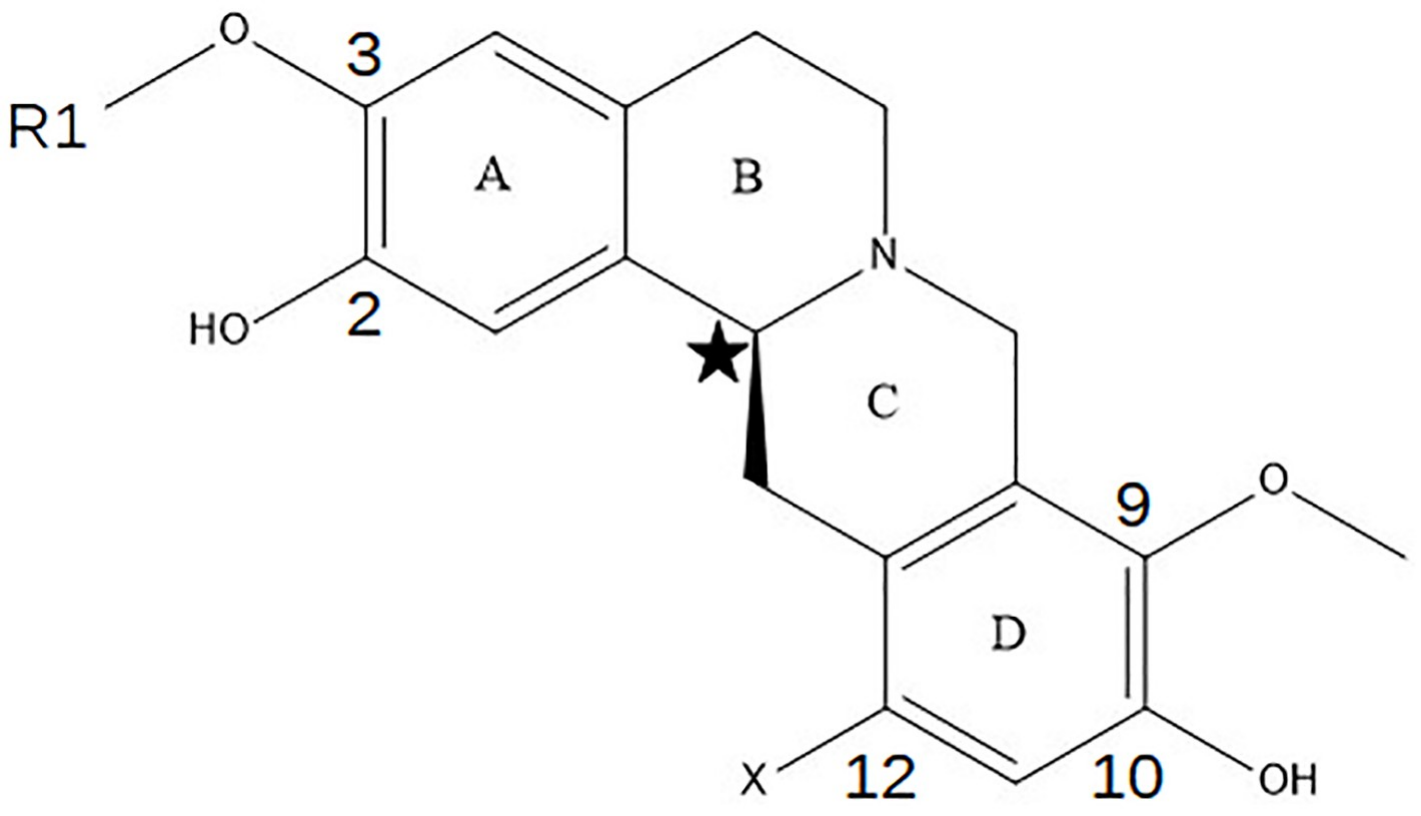
Structure of the (-)-stepholidine core with four rings annotated alphabetically as referenced in the text. R1 represents the substitution at the C3 position. The chiral carbon is labeled by a star.

In this study, we focus on the interaction of the D3 receptor with a series of derivatives of (-)-stepholidine (Table 1), a natural product displaying dual D1 and D2 activity and observed to have antipsychotic activities. [31, 38–40] Motivated by the previous work on the synthesis and activity of the (-)-stepholidine C9 derivatives [23] aimed at achieving a dual D1/D3 activity, we continued our Structure-Activity Relationship (SAR) studies using the tertrahydroprotoberberine (THPBs) scaffold to synthesize a new set of compounds targeting the dopamine receptors. In comparison to the compounds previously assayed which are substituted with alkyl chains at the C9 position of the THPB scaffold, compounds synthesized and studied in this work are substituted at the C3 position (Fig 2 and Table 1). The motivation of synthesis and substitution at the C3 position is to extend these molecules to access the secondary binding site (SBS) which have the potential to improve receptor selectivity for these compounds. [23] Due to the lack of a crystal structure, the mode of interaction of (-)-stepholidine derivatives with the D3 receptor remains uncertain. [23, 30, 41]

**Table 1:** List of the (-)-stepholidine derivatives considered in this work. All substitution are made at the C3 position of the (-)-stepholidine core as shown in Fig 2.

(-)-stepholidine C3 derivatives		
	x	R1
1a	H	Et
1b	H	n-Pr
1c	H	n-Bu
1d	H	n-Pen
1e	H	n-Hex
1f	H	2-fluoro ethyl

In this work, we report the first assessment of a novel computational strategy by using an implicit solvent model to model the effects of water expulsion in protein-ligand binding. This is done by acquiring the thermodynamic properties of binding site water molecules in dopamine D3 receptor from explicit solvent simulations and estimating the binding free energies of the complexes of (-)-stepholidine analogues’ with the D3 receptor by incorporating hydration parameters in an implicit solvent model. This allowed us to capture localized enclosed hydration effects which could not be captured by using conventional descriptions of solvation. Although limited to the Dopamine D3 receptor, this work is the first step in attempting to build a model of binding accurate enough to differentiate between sub-families of Dopamine receptors by exploiting potential differences in their hydration properties.

## Methods

### Hydration Site Analysis of the binding site of the D3 receptor

**Fig 3:**
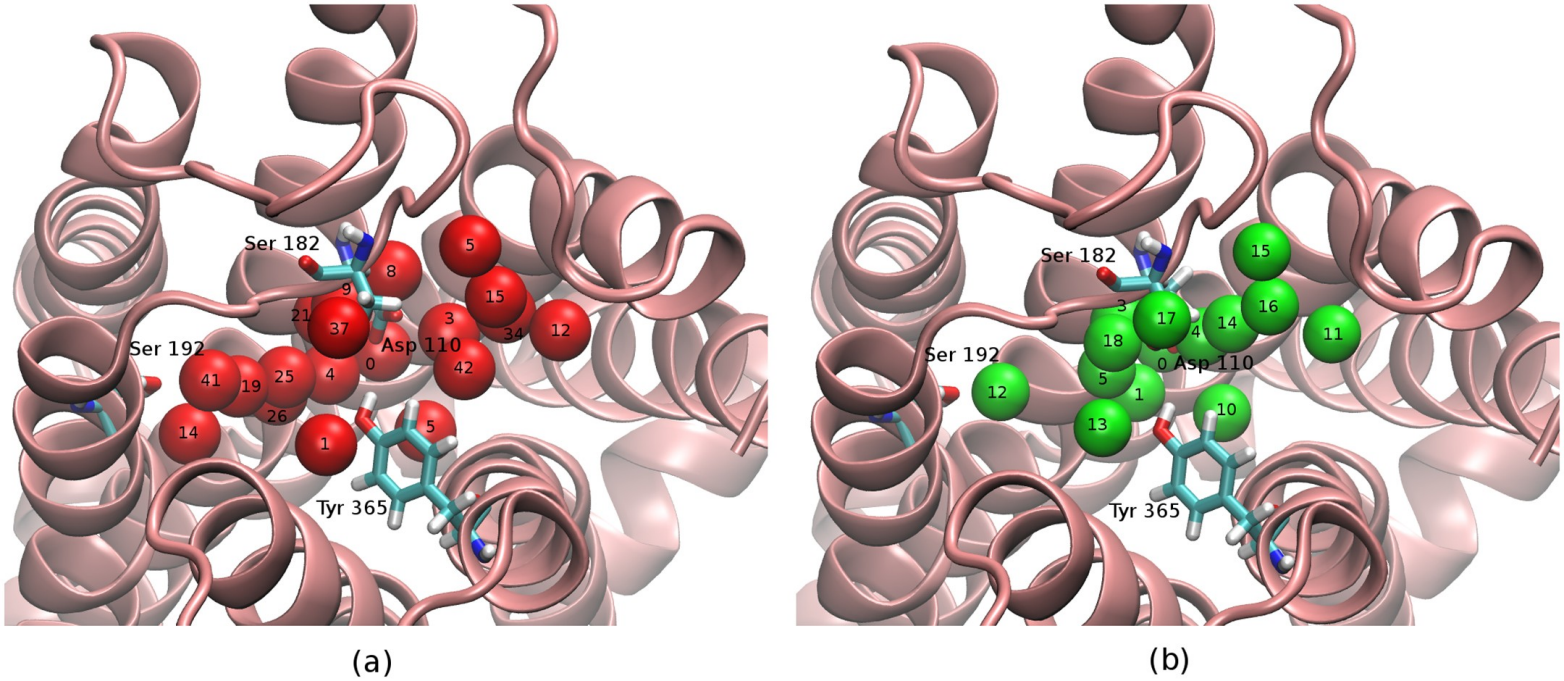
Hydration sites and corresponding AGBNP2 spheres at the dopamine D3 receptor binding site. (a) Location of hydration sites (red) within the binding cavity of the Dopamine D3 receptor as mapped by Hydration Site Analysis. (b) Hydration spheres (green) of the AGBNP2 model for the same receptor structure in (a). The positions of the AGBNP2 hydration spheres are functions of the internal coordinates of the receptor.

The thermodynamic and structural properties of water molecules in the binding site of the receptor were studied using the Hydration Site Analysis (HSA) method. [8, 11] Briefly, HSA is based on the analysis of molecular dynamics trajectories with explicit solvation, whereby molecular dynamics simulations are performed to identify regions with significant water density near the receptor surface. Average thermodynamic quantities such as enthalpy, entropy and free energies are calculated for these sites using the concept of Inhomogeneous Solvation Theory. [6, 42] HSA explicit solvent simulations are performed on a restrained receptor structure. The trajectories are then processed to cluster hydration site locations and analyzed for their thermodynamic estimates as described elsewhere. [6, 8] The total energy, *E*_total_ for each of these sites are calculated as the sum of the one-half of the mean solute-water *E*_sw_ interaction energy and one-half of the mean water-water *E*_ww_ interaction energy. The excess energies of the hydration sites relative to bulk value are used to classify them as either favorable or unfavorable water sites. Unfavorable sites are those that, when displaced by the ligand, are believed to enhance the binding affinity. The locations and average solvation energies for each of the sites identified for the D3 receptor are shown in Fig 3a and Table 2.

**Table 2:** Summary of the placement and parameterization of the AGBNP2 enclosed hydration spheres for the dopamine D3 receptor binding site.

Location^a^	HSA site Id^b^	AGBNP2 site Id^b^	AGBNP2 anchoring type ^c^	$p_{s}^{d}$	${\left(E-E_{\text{bulk}}\right)}^{e}$	$\left(E-E_{\text{bulk}}\right)\times p_{s}$ ^f^
OBS	0	0,1	Asp 110 backbone carbonyl	1.00	2.36	2.36
OBS	3,4,8,21	3,4,5	Asp 110 side chain carboxylate	0.86	3.28	2.83
OBS	25,26	9	Center of mass	0.66	1.92	1.27
OBS	14,19,41	12	Center of mass	0.57	5.32	3.05
OBS	1	13	Center of mass	0.87	2.80	2.44
OBS/SBS boundary	11	10	Center of mass	0.83	2.32	1.92
OBS/SBS boundary	9	18	Ser 182 hydroxyl hydrogen	0.87	0.21	0.19
SBS	12	11	Center of mass	0.63	0.92	0.58
SBS	34,42	14	Center of mass	0.58	1.31	0.77
SBS	5	15	Center of mass	0.95	0.34	0.33
SBS	15	16	Center of mass	0.68	0.50	0.34
SBS	37	17	Center of mass	0.34	0.08	0.03

^*a*^OBS: Orthosteric binding site; SBS: Secondary binding site.

^*b*^Ste Id as shown in Fig 3

^*c*^See reference.

^*d*^Average water occupancy of the site measured by HSA

^*e*^Average energy of the site relative to bulk measured by HSA, *E*_bulk_ = −12.24 kcal/mol.

^*f*^Overall energy score of the HSA sites indicated in column 2 and of the enclosed hydration score of the AGBNP2 hydration spheres indicated in column 3 in kcal/mol.

Proteins can be highly dynamic. Hence, a single structure is often an insufficient representation of the structural variability of the hydration layer of a protein receptor. This is particularly so in the present work, where the ligands we considered could induce different conformations of the receptor when bound. To address conformational variability, in this work, we obtained HSA hydration maps for a series of D3 receptor structures obtained from induced-fit docking calculations with different ligand types, which included the previously reported C9-substituted ligands [23], and the available crystal structure [28] (see Computational Details). The location and energies of the hydration sites were averaged from all receptor conformations to obtain a single hydration map as shown in Fig 3a.

The solvation energies and locations of the explicit hydration sites were then used to position the first-shell hydration spheres of the AGBNP2 (Analytical Generalized Born Non Polar) implicit solvation model [20] and to set their strengths (see below). The strength of the hydration spheres were set according to the HSA scores

$$\begin{array}{c} {}[E_{\text{total}}\left(i\right)-E_{\text{bulk}}]p\left(i\right) \end{array}$$ (1)

where *i* is the index of the HSA hydration sites, *p*(*i*) is the water occupancy of the site, *E*_total_(*i*) is the total energy of the site and *E*_bulk_ is the corresponding reference value obtained from OPC [43] neat water (*E*_bulk_ = −12.24 kcal/mol).

### Parameterization of the AGBNP2 enclosed hydration model

Even slight variations in atomic positions are known to cause significant changes in hydration structure. [10, 44, 45] We attempted to capture specific ligand-induced conformational changes, as well as thermal fluctuations of the hydration structure by considering multiple structures of the D3 receptor (see Computational Details). Hydration site maps were obtained individually for each of the three receptor structures using HSA. [8] These hydration maps were then integrated into a single hydration map (see Fig 3a) by averaging the free energy weights of neighboring hydration sites from the individual maps. The energies and water occupancies of the HSA hydration regions were used to obtain the enclosed hydration corrections for the AGBNP2 first-shell hydration spheres using Eq (1) (see below).

**Fig 4:**
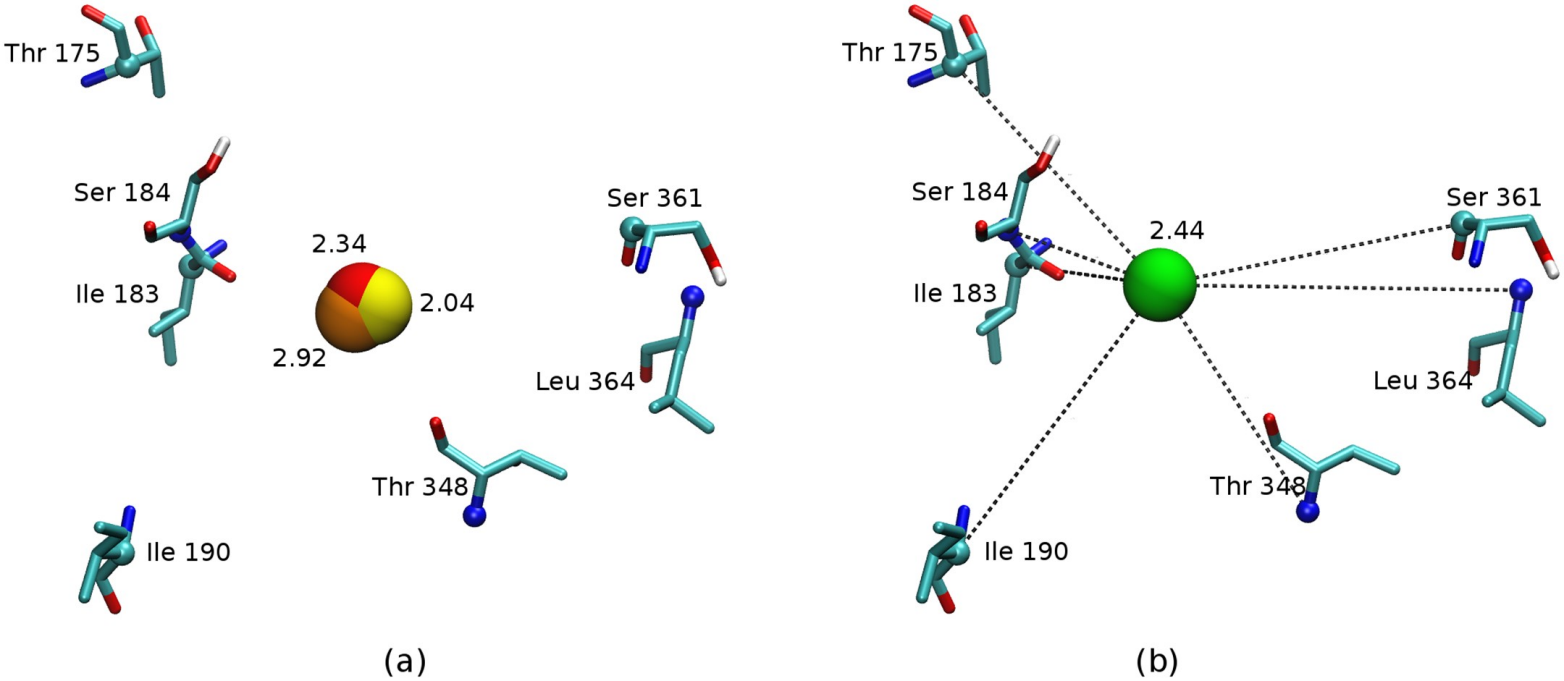
Strategy for scoring and placement of AGBNP2 hydration spheres in dopamine D3 receptor binding site. (a) Location of a hydration site identified by HSA using three receptor structures (residues from one receptor structure shown for clarity); the overlapping red, yellow and orange spheres represent a hydration site identified by each receptor structure; the energetic penalties incurred from each HSA map are annotated in kcal/mol, (b) An AGBNP2 hydration sphere (green) is placed and scored by averaging the energetic penalties from the three maps at the location of the HSA site; the AGBNP2 hydration sphere is placed at the geometrical center of the atoms represented in CPK and is anchored to respective atoms during the simulation.

The energetically unfavorable hydration sites identified by HSA, and thus good candidates for displacement by the ligand, were found to be distributed throughout the dopamine D3 receptor binding site. These were reproduced as best as possible with AGBNP2 first-shell hydration spheres within the limitations of the available anchoring methods. [9, 20] To ensure translational and rotational invariance of the AGBNP2 implicit solvation function, hydration spheres are located only in terms of molecular internal coordinates, that is by specifying distance and angle geometries in relation to selected atoms of the receptor. The geometries that were employed most often in this work have been for sites attached to polar hydrogen atoms and for sites anchored to mimic the lone pair orbitals of carbonyl and carboxylate groups. When a suitable anchoring geometry could not be found, AGBNP2 hydration spheres have been positioned at the geometrical center of a group of atoms of the receptor, typically backbone C_*α*_, C_*β*_ and N atoms (Fig 4). [9] The resulting AGBNP2 first-shell hydration spheres are shown in Fig 3b and their parameterization are listed in Table 2.

Because of the complexities of enclosed hydration phenomena and their variations due to the motion of receptor atoms, it has been challenging to formulate an unsupervised and automated protocol to map HSA results to AGBNP2 spheres. Within the general framework outlined above, some manual adjustments were made. One adjustment was made to model strongly unfavorable HSA hydration sites (HSA site Ids—3,4,8 and 21) identified at hydrogen-bonding distance to the carboxylate group of the critical Asp110^3.32^ residue. Because AGBNP2 attaches eight equinergetic solvation spheres to carboxylate groups, [20] we decided to distribute the HSA excess energy of this site among the three out of eight carboxylate hydration spheres of Asp110^3.32^ with non-zero water occupancy. Adjustments were also made to treat HSA hydration sites in close proximity to each other. Due to the limitations in mapping accurately the position of AGBNP2 spheres, in these case, we modeled nearby groups of HSA sites with a single AGBNP2 hydration sphere by assigning to it the sum of the energy weights of each HSA site as shown in Table 2.

### Binding free energy model

The protein-ligand complexes are modeled using the OPLS-AA/AGBNP2 effective potential, in which the OPLS-AA [46, 47] force field defines the covalent and non-bonded inter-atomic interactions. Solvation effects are modeled implicitly using the Analytic Generalized Born plus non-polar (AGBNP2) model. [20] According to this model, the hydration free energy Δ*G*_h_ of the receptor-ligand complex is computed as the sum of electrostatic Δ*G*_elec_, non-polar, Δ*G*_np_, and short-range solute-water interactions, Δ*G*_hs_:

$$\begin{array}{c} \Delta G_{h}=\Delta G_{\text{elec}}+\Delta G_{\text{np}}+\Delta G_{\text{hs}} \end{array}$$ (2)

The electrostatic component of the hydration free energy is computed using a modified continuum dielectric Generalized Born model. [48, 49] The non-polar component includes a surface-area dependent term that accounts for the free energy of creating the solute cavity within the solvent, and a Born-radius dependent term that accounts for long range solute-solvent van der Waals interactions. [20] In AGBNP2, short-ranged solute-solvent interactions, such as hydrogen bonding are modeled by means of hydration spheres placed on the solute surface. A geometrical procedure measures the water occupancy of each hydration sphere, which is then used to weigh its contribution to the solute hydration free energy according to the expression:

$$\begin{array}{c} \Delta G_{\text{hs}}=\sum_{s}h_{s}S\left(w_{s}\right) \end{array}$$ (3)

where *w*_*s*_ is the water occupancy factor of the sphere defined as

$$\begin{array}{c} w_{s}=\frac{V_{s}^{\text{free}}}{V_{s}} \end{array}$$ (4)

where *V*_s_ is the volume of each sphere and $V_{s}^{\text{free}}$ is the volume of the portion of the sphere occupied by water. *S* is a switching function that smoothly turns off an hydration sphere if its water occupancy is below a given threshold. The *h*_*s*_ parameter measures the hydration strength of the corresponding hydration site. Negative *h*_*s*_ values describe hydration sites contributing favorably to the hydration free energy, whereas positive values are used for sites which contribute unfavorably to the hydration free energy. [9]

In this study, almost all hydration sites identified by HSA inside the binding site are energetically unfavorable. The strength of AGBNP2 hydration site spheres, thus having positive *h*_*s*_ values are used to define unfavorable water molecules in the binding site of the receptor, which, when displaced by the ligand, contribute favorably to binding. The *h*_*s*_ energy values are obtained from the explicit solvent HSA analysis as described above and are listed in Table 2.

Absolute binding free energies of the dopamine D3 receptor bound to (-)-stepholidine C3 analogues were calculated by means of a Single Decoupling (SDM) binding free energy approach [50] employing an alchemical potential energy function of the form:

$$\begin{array}{c} U_{\lambda }\left(r\right)=U_{0}\left(r\right)+\lambda u\left(r\right) \end{array}$$ (5)

where **r** = (**r**_R_, **r**_L_) are the atomic coordinates of the receptor-ligand complex, *U*_0_ represents the effective potential energy of the uncoupled complex when receptor and ligand are not interacting (such as if they were at infinite separation), λ is the alchemical progress parameter which linearly couples receptor and ligand through the binding energy function *u*(**r**), defined as the change in the effective potential energy of the complex for bringing the receptor and ligand from infinite separation to the conformation **r**. Based on Eq (5), the complex is uncoupled at λ = 0 and coupled at λ = 1. The free energy difference between these two states is defined as the excess free energy of binding, Δ*G*_*b*_. [51]

The binding free energy calculation protocol entails simulating the system at series of λ values spaced between 0 and 1 and collecting binding energy samples at each state. The binding energy values from each λ state are then processed using UWHAM [52] to obtain the excess free energy of binding Δ*G*_*b*_ and corresponding uncertainty. The standard free energy of binding $\Delta G_{b}^{{}^{\circ}}$ is obtained by adding the concentration and binding site volume term to the excess free energy (see Computational Details).

Average interaction energies Δ*E*_b_ for analysis are obtained by averaging the binding energy values of the complexes from the ensemble of conformations at the bound state at λ = 1. The uncertainties of binding energy values are estimated from the standard error of the mean. The reorganization free energies for binding, defined as $\Delta G_{\text{reorg}}^{{}^{\circ}}=\Delta G_{b}^{{}^{\circ}}-\Delta E_{b}$, are obtained from the corresponding values of the standard binding free energy and of the binding energy. The uncertainty of the reorganization free energy is obtained by standard error propagation.

As an alternative to simulating each alchemical λ state independently, to accelerate the convergence of free energy calculations, in this work we utilize an Hamiltonian replica-exchange approach [53, 54] where λ values are exchanged between molecular dynamics replicas, allowing the mixing of intermolecular degrees of freedom to explore the conformational space efficiently. [53]

### Computational details

#### Hydration Site Analysis (HSA) in explicit solvent

Three D3 representative receptor structures were used for the Hydration Site Analysis (HSA) in explicit solvent. The receptor structures considered are those corresponding to the complexes of D3 with (-)-stepholidine, C3 butyl (**1c**) and C9 butyl derivatives [23] as obtained from individual induced fit docking (IFD) simulations [55] using the crystal structure receptor configuration of the dopamine D3 receptor (PDB ID—3PBL) as a starting point. The IFD protocol was performed in five steps: generation of ligand conformations, initial docking with reduced receptor atom van der Waal radii, side chain minimization with Prime [56, 57], a second docking step using the new receptor configuration and finally pose scoring. Receptor-ligand configuration with the highest IFD score ranking was selected, except in the case where the highest scored pose did not maintain the well conserved Asp 110^3.32^ salt-bridge. The apo receptor structure from each highest scored pose, was then used for Hydration Site Analysis (HSA).

The explicit solvent simulations for Hydration Site Analysis (HSA) were conducted with the AMBER [58] software package with the OPC [43] water model with positional restraints on all heavy atoms with a force constant of 10.0 kcal/mol/Å^2^. Each system was minimized and thermalized for 2.0 ns under NPT conditions of 1 atm and 300K. During the production run, MD simulations were performed for 10.0 ns under NVT conditions and snapshots of the trajectory were collected every 1.0 ps. High density spherical regions of 1Å radius were identified using a clustering analysis on the water molecules which lies within 8 Å of the superimposed ligand in D3 binding site. Individual hydration sites were then populated with all water molecules that lies within 1 Å of the corresponding hydration site center. Average solvation energies were calculated for each site by calculating the energies of the water molecules within 1 Å of each hydration site center in all 10,000 frames of the trajectory. For technical reasons, HSA employed a different force field (AMBER ff14SB force field [59]) than that for the binding free energy calculations (OPLS/AA). The purpose of HSA is to obtain semi-quantitative estimates of the energies of enclosed water molecules as well as their locations. On a qualitative level, The large increase of binding affinities when including enclosed hydration effects (observed below) is not expected to depend on the choice of the force field.

#### System preparation for the binding free energy calculations

The bound ligand was removed from the co-crystallized structure of Dopamine D3 receptor with eticlopride [28] along with crystallographic waters. Protonation states were adjusted to reflect neutral pH conditions. The receptor structure was prepared using the Protein Preparation Wizard of the Maestro version 2016-3 (Schrodinger Inc.). The prepared protein structure was used to generate the receptor grid for docking using default parameters. Docking was performed with Standard Precision (SP) version of the Glide program (Schrodinger Release 2016-3). [60] Positional constraints were applied to the alkyl nitrogen of the (-)-stepholidine and all the analogues to maintain the salt-bridge interaction with Asp 110^3.32^ of the D3 receptor. The hydroxyl and thiol groups of the receptor, such as of residues Ser 182^ECL2^, Ser 192^5.42^, Ser 196^5.46^, Thr 115^3.37^, Thr 369^7.39^, Cys 114^3.36^ located near the binding site were allowed to rotate during docking.

The (-)-stepholidine C3 analogues were built using the Maestro program (Schrodinger Release 2016-3). Alternative protonation states as well as chiral forms were generated for the 7 ± 2 pH range using the LigPrep facility (Schrodinger Inc.) and ionization penalties were calculated with Epik [61] at pH 7. The ionization free energies were recorded and added to the binding free energy estimates to compute the predicted binding free energies. Only states where the alkyl nitrogen is protonated were selected for docking calculations. We also included in the docking study the two chiral forms of the protonated alkyl nitrogen for each compound as generated by LigPrep (Schrodinger Release 2016-3).

Binding poses generated by docking were selected based on their docking scores and presence of an ionic interaction between the protonated alkyl nitrogen and the carboxylate group of Asp110^3.32^. The derivatives considered here are all stereoisomers with the S configuration at the chiral carbon connecting ring B and ring C of the (-)-stepholidine core (Table 1). The adjacent protonated alkyl nitrogen atom is found always in the S configuration while maintaining the salt-bridge interaction.

The starting conformations of complexes from docking underwent energy minimization and thermalization. Hamiltonian Replica-exchange Molecular dynamics simulations were performed starting from the thermalized structures using 28 intermediate lambda states distributed as follows: 0.0, 0.002, 0.005, 0.008, 0.009, 0.01, 0.0105, 0.012, 0.0135, 0.015, 0.02, 0.0225, 0.025, 0.03, 0.035, 0.04, 0.07, 0.1, 0.25, 0.35, 0.45, 0.55, 0.65, 0.71, 0.78, 0.85, 0.92, and 1.0. The volume of the binding site, *V*_site_ is defined as the spherical volume in which the center of mass of ligand is within 3.5 Å of the center of mass of the binding site of the D3 receptor, defined as the center of mass of the C_*α*_ atoms of the residues 110, 111, 114, 183, 188, 346, 349 and the C_*β*_ atoms of residues 342, 349 and the backbone nitrogen atom of residue 111. The binding site volume restraint is implemented as a flat-bottom spherical harmonic potential with force constant of 3 kcal/mol/Å^2^ and tolerance of 3.5 Å which resulted in a free energy penalty $\Delta G_{t}^{{}^{\circ}}$ for transferring the ligand from a solution of concentration *C*° to a volume of size *V*_site_, of about 1.32 kcal/mol, calculated from the following expression:

$$\begin{array}{c} \Delta G_{t}^{{}^{\circ}}=-k_{B}TlnC^{{}^{\circ}}V_{\text{site}} \end{array}$$ (6)

The receptor conformation was loosely restrained to the crystallographic structure using flat-bottom positional restraints with a force constant of 25 kcal/mol/Å^2^ and a tolerance of 1.5 Å applied to the backbone C_*α*_ atoms, except for six residues 180-185 of the ECL2 loop to account for its flexibility.

Temperature replica-exchange simulations were carried out to obtain conformational reservoirs of the apo receptor. [62] These utilized 23 replicas distributed between 300 and 400K. [62] The conformational ensemble collected at 300K was used as a source of apo-receptor conformations in the replica-exchange simulations. Conformational reservoirs for each ligand were generated similarly using 8 replicas distributed between 300 and 600K. During the simulation, conformations of receptor and ligands were randomly selected from the conformational reservoirs during exchanges at the fully uncoupled state.

Single-decoupling binding free energy calculations were performed for approximately 1 ns per replica for a total of 28 ns per complex. Binding energies samples from the last 500 ps were used for the binding free energy estimates. Each cycle of replica lasted 10 ps with 1 fs MD time-step. Binding energies were collected every 10 ps. Most of the calculations were carried out at the XSEDE SuperMIC and Stampede2 clusters utilizing CPU’s and MIC devices.

To improve the convergence of the binding energies near the uncoupled state at λ = 0, we employ a soft core binding energy function as described elsewhere. [52, 63] The binding energies were analyzed using the UWHAM R-statistical package [52] to yield the binding free energy $\Delta G_{\text{b}}^{{}^{\circ}}$. As mentioned, the average interaction energy Δ*E*_b_ of each complex was obtained from the value of the average binding energy at the coupled state (λ = 1). Reorganization free energies $\Delta G_{\text{reorg}}^{{}^{\circ}}$ were measured as the difference between the binding free energy and the average binding energy as $\Delta G_{\text{reorg}}^{{}^{\circ}}=\Delta G_{b}^{{}^{\circ}}-\Delta E_{b}$.

#### Synthesis and experimental assays of (-)-stepholidine C3 analogues

Compounds **1a**-**1f** were synthesized using the procedure developed as shown in Fig A and described in S1 File. Commercially available dihydroxy benzaldehyde, **4** was selectively protected with a benzyl group to give compound **5**. Second, the phenolic group of aldehyde **5** was protected with a silyl group and the intermediate was subjected to a Henry condensation reaction to give nitrostyrene **6**. Reduction of nitro compound **6** using LiBH_4_ yielded primary amine **7**. Aminolysis of lactone **8** with primary amine **7** was carried out to give amide alcohol **9**, which was acetylated to afford **10**. Ring B of the tetrahydroprotoberberine (THPB) scaffold was formed via Bischler-Napieralski cyclization followed by asymmetric hydrogenation using Noyori’s catalyst and formic acid/triethylamine mixture to generate **11** with good yield (88%). Hydrolysis of the acetyl group and subsequent chlorination endowed us the tetracyclic scaffold of THPB in compound **12**. The enantiomeric excess of this common precursor was found to be 90.2% (chiral HPLC) and it was used for further analogue generation. Alkylation of compound **12** followed by debenzylation provided us the C3 analogues **1a**-**1f**.

All the (-)-stepholidine C3 analogues were biochemically evaluated by primary and secondary radioligand binding assays with the dopamine receptor to obtain the inhibition constants of binding, *K*_*i*_ and reported in Table 3. Both the primary and secondary radioligand binding assays were done at the PDSP facility (http://pdsp.med.unc.edu/). In the primary binding assays, compounds were tested at single concentrations (10 μM) in quadruplicate in 96-well plates. Compounds that showed a minimum of 50% inhibition at 10 μM were tagged for secondary radioligand binding assays to determine equilibrium binding affinity at specific targets. In the secondary binding assays, selected compounds were tested in triplicate sets (3 sets of 96-well plates) at eleven different concentrations out of which eight are in nanomolar range (0.1, 0.3, 1, 3, 10, 30, 100 and 300 nM) and rest of the three concentration in micromolar range (1, 3, and 10 μM). Both primary and secondary radioligand binding assays were carried out in a final of volume of 125 μl per well in appropriate binding buffer. The hot ligand concentration was usually at a concentration close to the *K*_*d*_ (unless otherwise indicated). Total binding and nonspecific binding were determined in the absence and presence of 10 μM Chlorpromazine, which was used as a reference compound. In brief, plates were usually incubated at room temperature and in the dark for 90 min. Reactions were stopped by vacuum filtration onto 0.3% polyethyleneimine (PEI) soaked 96-well filter mats using a 96-well Filtermate harvester, followed by three washes with cold wash buffer. Scintillation cocktail was then melted onto the microwave-dried filters on a hot plate and radioactivity was counted in a Microbeta counter. For detailed experimental details, please refer to the PDSP website http://pdsp.med.unc.edu/ and click on ‘Binding Assay’ or ‘Functional Assay’ on the menu bar.

**Table 3:** Measured inhibition constants of binding (*K*_*i*_) for the (-)-stepholidine C3 analogues against the dopamine D3 receptor.

Compounds	C3-substituent	*K_i_*^a,b^
1a	Et	40.0
1b	n-Pr	46.0
1c	n-Bu	51.0
1d	n-Pen	33.0
1e	n-Hex	26.0
1f	2-fluroethyl	86.0

^*a*^ In nM. Experiments were carried out in triplicate—uncertainties are estimated as 13% of reported *K*_*i*_;

^*b*^[^3^H] N-methylspiperone used as radioligand; chlorpromazine used as a reference compound with *K*_*i*_ = 11.0 nM. The biochemical details of the assay are provided in the main text.

## Results

### Biochemical evaluation of (-)-stepholidine C3 analogues

The inhibition constants for binding of the C3 analogues are reported in Table 3. The C3 analogues showed relatively stronger inhibition of binding at the dopamine D3 receptor compared to that of C9 analogues tested previously. [23] The length of the C3 substitution has generally a small influence on their measured affinities in this set. However, the analogues with the longest C3 pentyl and hexyl substituent (**1d** and **1e**) exhibit a slightly stronger affinity (Table 3).

### Binding free energy calculations

We employed the enclosed hydration model described above to study six derivatives of (-)-stepholidine substituted at the C3 position with and without the enclosed hydration corrections to probe the effects of enclosed hydration on the binding free energy predictions (Table 4).

**Table 4:** Experimental and calculated binding free energies, average binding energies and reorganization free energies of the (-)-stepholidine C3 analogues with and without enclosed hydration corrections.

Compound	$\Delta G_{\text{exp}}^{{}^{\circ}}$^a,b^	Without enclosed hydration model			With enclosed hydration model		
		$\Delta G_{\text{calc}}^{{}^{\circ}}$^b,c^	Δ*E*_b_^b,c^	$\Delta G_{\text{reorg}}^{{}^{\circ}}$^b,c^	$\Delta G_{\text{calc}}^{{}^{\circ}}$^b,c^	Δ*E*_b_^b,c^	$\Delta G_{\text{reorg}}^{{}^{\circ}}$^b,c^
1a	−10.1	−2.2	−36.9	34.7	−8.8	−42.5	33.7
1b	−10.0	−2.3	−38.0	35.7	−10.4	−44.7	34.3
1c	−10.0	−1.8	−40.3	38.5	−11.5	−48.1	36.6
1d	−10.2	−0.3	−43.7	43.4	−10.6	−55.6	45.0
1e	−10.4	−3.9	−39.6	35.7	−12.5	−55.2	42.7
1f	−9.6	−3.1	−32.7	29.6	−8.9	−43.2	34.3
RMSE^b,d^		7.9			1.2		
Correlation coefficient (r)		−0.014			0.64		

^*a*^ Experimental affinities are calculated using the relation $\Delta G_{\text{exp}}^{{}^{\circ}}=k_{B}T\;lnK_{i}$ where *K*_*i*_ is the inhibition constant of binding, *k*_*B*_ is the Boltzmann’s constant.

^*b*^In kcal/mol.

^*c*^Approximate uncertainties for all measurements are implied by the number of significant figures; the actual values of the uncertainties for each measurement are provided in Table A in S1 File.

^*d*^Root mean square error relative to the experimental binding free energies.

**Fig 5:**
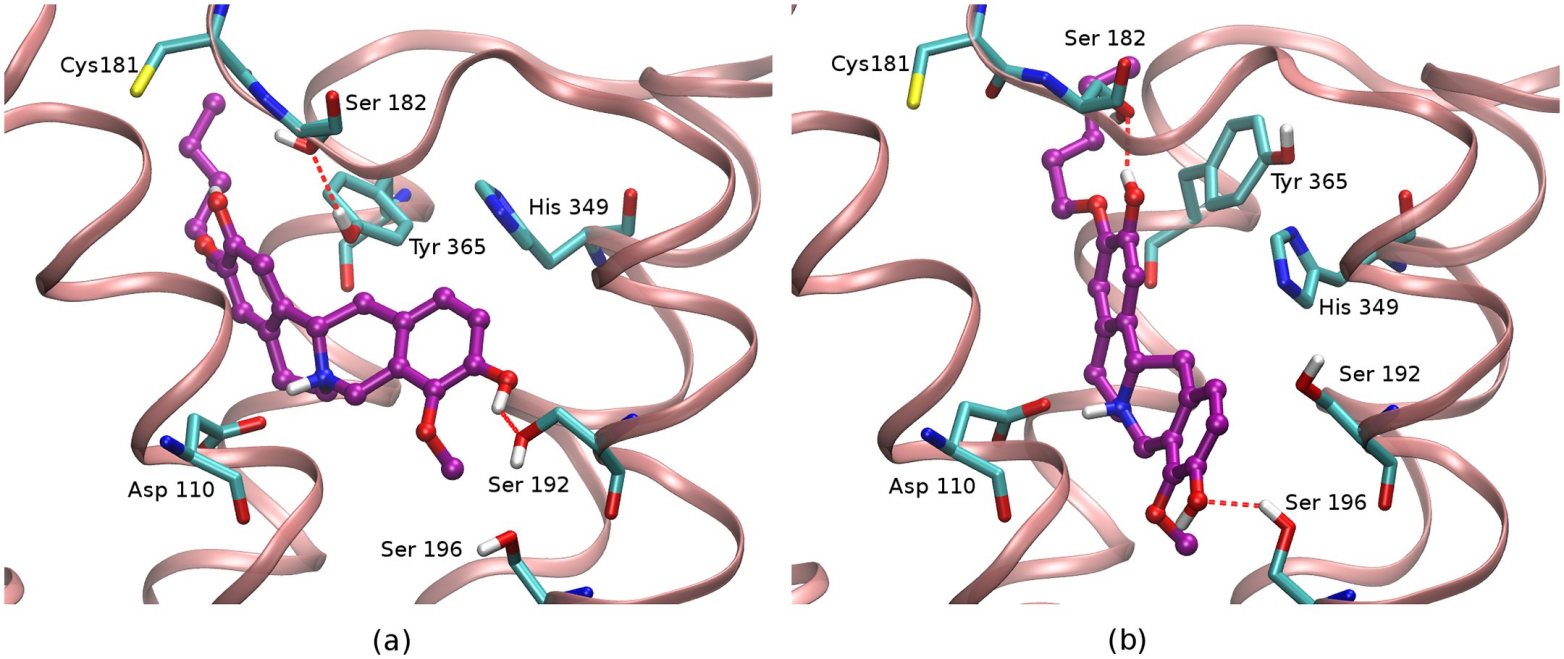
Interactions of C3 pentyl analogue with the dopamine D3 receptor. a) The C3 pentyl analogue (3e, purple) of (-)-stepholidine is observed to interact with Ser 192 of the receptor at the orthosteric binding site. In order for the C3 analogues to interact with Ser 192, the C10 hydroxyl group is placed in proximity of Ser 192; b) The 3e C3 analogue in another observed binding pose in which it interacts with Ser 196, rather than Ser 192. In this pose, ring D of the (-)-stepholidine core is bound deeper into the orthosteric binding site and the ligand is twisted causing Tyr 365 in the SBS to rotate and move away from Ser 182 of ECL2. The receptor is represented as a pink ribbon.

The (-)-stepholidine C3 analogues are substituted at the third position of ring A of the (-)-stepholidine core. To accommodate the long alkyl chain substituents, the C3 analogues (Fig 5) are found to dock to the dopamine D3 receptor in a binding pose so that the alkyl chain occupies the secondary binding site (SBS). This has the important consequence that ring D, occupies the OBS so to maintain the salt bridge with Asp 110^3.32^ in contrast to C9 analogues where ring A occupy the OBS [23].

The enclosed hydration model is found to be an essential ingredient to reproduce the observed affinities. Binding free energy estimates of C3 derivatives obtained without enclosed hydration grossly underestimate the magnitudes of the experimental affinities derived from the measured inhibition constants of binding (Table 4, 2nd and 3rd columns). In contrast, binding free energy calculated with the enclosed hydration model are significantly more favorable and substantially in better quantitative agreement with the experiments than without enclosed hydration (Table 4, 2nd and 6th columns). When employing the enclosed hydration model, the root mean square error (RMSE) is reduced by a factor of 6 and, while variations in the experimental values are slight (Table 4, 2nd column), the level of correlation increased from less than zero to 64%. The values of the calculated binding free energies with enclosed hydration are all within 2 kcal/mol of the experiments.

## Discussion

Though efficient and faster convergence of binding free energy calculations can be achieved using implicit solvent models, these lack the ability to model solvent heterogeneity and confinement in molecular simulations, especially within deep protein binding pockets. In absence of ligand, enclosed water molecules form network of interactions among themselves and with receptor atoms, which are fundamentally different from those present in the bulk and solvent exposed regions of the protein. [8, 11] Water molecules which maintain favorable contacts with the protein or act as bridging waters generally disfavor binding when displaced by the ligand. However, energetically and entropically frustrated water molecules such as those trapped within the hydrophobic regions of the binding site, favor binding when displaced by the ligand. In this work, we have employed for the first time a hybrid computational model involving explicit and implicit solvation to include the thermodynamics of confined water in the calculation of the binding free energies of protein-ligand complexes. We applied the model to calculate the binding free energies for a series of novel compounds as potential ligands of the dopamine D3 receptor, which have been synthesized and assayed for activity as part of this work. In all cases tested, binding free energies were observed to be more favorable in the presence of enclosed hydration effects compared to the conventional implicit solvent model. The enhancement of binding affinities with the enclosed hydration model is in accord with the idea that energetically frustrated enclosed water molecules contributed favorably to binding when displaced by the ligand.

In this study, we identify a class of dopamine D3 receptor ligands which are more powerful than those previously synthesized and assayed. [23] The affinities of the (-)-stepholidine C3 analogues, synthesized in this work, justifies the motivation of synthesis to increase interaction at the secondary binding site (SBS) by adding substituents at the C3 position, with the strongest affinitiy being observed for the longest substitution (**1e**) in agreement with the computational predictions (Table 3). The modeling approach introduced here has provided key insights for this system. All of the compounds analyzed consistently maintained an ionic interaction between the protonated alkyl nitrogen of the (-)-stepholidine core and the carboxylate group of Asp110^3.32^ of the D3 receptor.

**Fig 6:**
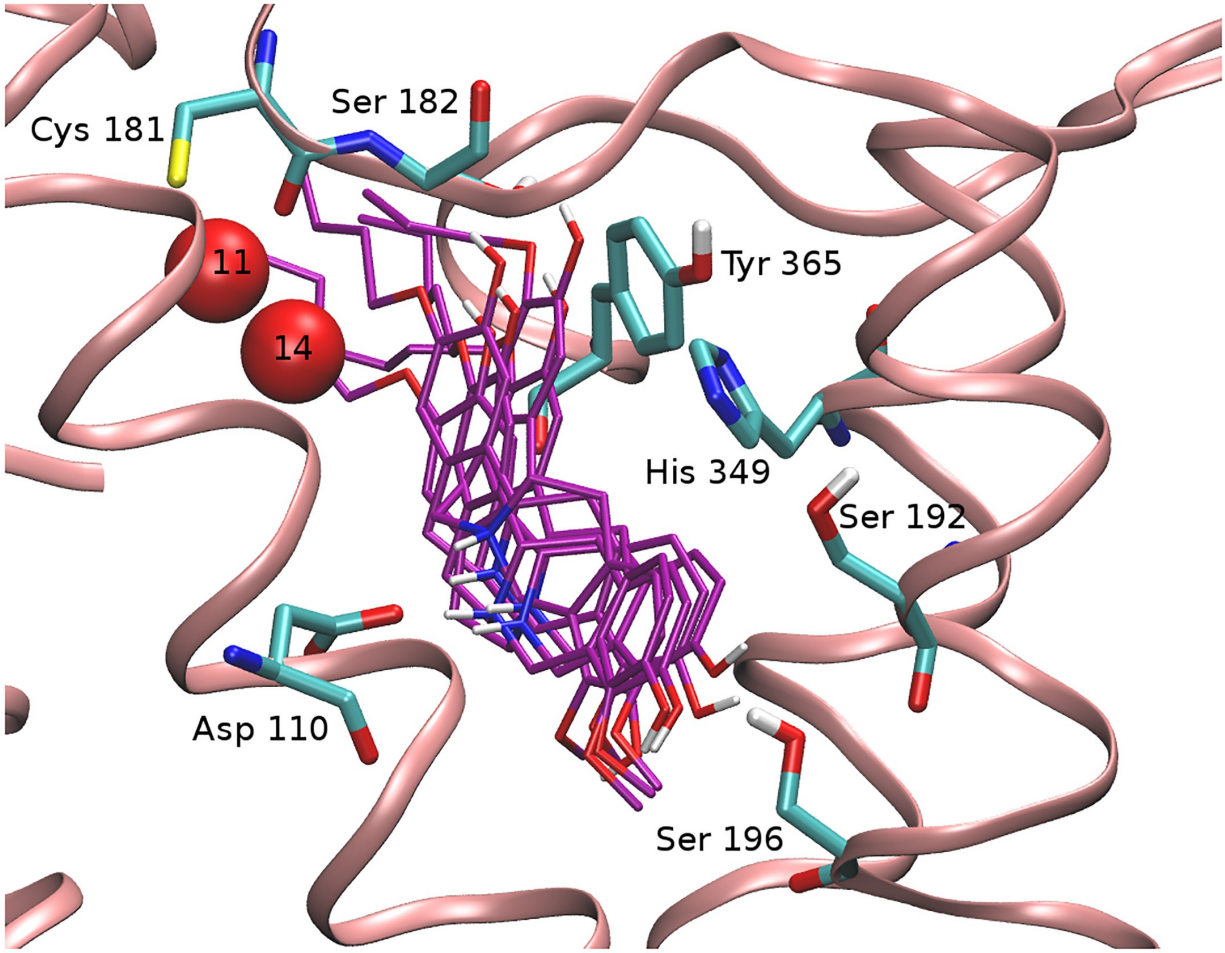
Displacement of enclosed water molecules by the (-)-stepholidine C3 analogues. Representative bound poses of (-)-stepholidine C3 analogues (purple) interacting with Ser 196 at the orthosteric binding site of the dopamine D3 receptor is observed to displace fewer enclosed water molecules, especially at the secondary binding site. AGBPN2 sites 11 and 14 are not displaced in this conformation of the C3 analogues (Table 2).

The positioning of C3 analogues within the binding site affect not only the pattern of ligand-receptor interactions in the secondary binding site, but crucially, also the interactions within the orthosteric pocket as well as the pattern of displacement of energetically unfavorable water molecules (Fig 6). These energetic and structural features are ultimately reflected in the differences of binding affinities with and without enclosed hydration effects (Table 4). When not considering enclosed hydration effects, the calculated binding affinities of the C3 analogues are observed to be very overly unfavorable. Inclusion of the enclosed hydration effects in the calculation, made the calculated binding free energies more favorable and improved the agreement with the experimental values (Table 4).

In our model, ring D of the (-)-stepholidine C3 analogues is placed into the orthosteric binding pocket where it is observed to interact with Ser 192^5.42^ through one hydrogen bond interaction with the hydroxyl group at position C10. In addition, the hydrogen bond interaction of C3 analogues is not stably maintained throughout the simulation, as it is seen to periodically switch to an alternate hydrogen bonding interaction with Ser 196^5.46^ slightly deeper into the orthosteric binding site (Fig 5b). Also, the binding of C3 analogues is observed to displace almost all enclosed water molecules within the orthosteric binding site by placing the (-)-stepholidine core. However, while interacting with Ser 196^5.46^, the alkoxy substituent chain at the secondary binding site (SBS) displaced fewer enclosed water molecules. These enclosed water sites, however, impose less energetic penalties, totaling to less than 1.5 kcal/mol (sites 11 and 14, see Table 2 and Fig 6), thereby contributing to little difference in the calculated binding affinities between the C3 derivatives. Another interesting observation in this pose is the displacement of Tyr 365^7.35^ of helix VII away from the secondary binding site (Fig 5b) and the concurrent disruption of the hydrogen bond interaction with Ser 182^ECL2^ which stabilizes the extracellular loop 2 (ECL2) in the SBS.

Conformational changes within the binding site may change the number and pattern of ligand-receptor interactions [64] as well as the hydration structure, which we know to be very sensitive to the placement of receptor atoms. While the use of AGBNP2 hydration spheres to model enclosed hydration is likely of general applicability, the specific parameterization used in this work is limited to the Dopamine D3 receptor. All calculations were done in absence of the description of the cellular membrane while limiting large backbone motions. Despite these limitations, our computational protocol was able to correctly predict the affinities of the C3 analogues with reasonable accuracy.

All these observations illustrates the complexities associated with binding of the (-)-stepholidine analogues to the dopamine D3 receptor. They also underscore the challenges encountered in the design of effective and selective D3 ligands/antagonists. [21, 23, 25, 35, 37, 65] One major challenge is the effect of the specific remodeling of the receptor binding site induced by ligands. In our study, induced fit docking calculations have not revealed major structural changes for different (-)-stepholidine analogues, although Hydration Site analysis (HSA) revealed more significant changes in the hydration energies and location of the hydration sites. The modeled binding affinities of the C3 analogues in this work may reflect the limitations imposed by the initial receptor structure. Another computational challenge in this work has been the appropriate representation of the enclosed hydration sites by exploiting the available topologies afforded by the current AGBNP2 implicit solvent model.

## Conclusion

In this study, we exploited the energetics of confined water molecules as obtained from explicit solvent simulations, and trained an implicit solvent model to account their effects on protein-ligand binding free energies, using a hybrid approach which proved useful for host-guest binding thermodynamics. [9]

Protein binding sites are much more complex than host-guest systems both in terms of structure and conformational variability. This is the first report of the implementation of a hybrid explicit-implicit solvent approach to calculate the binding affinities of protein ligand complexes and its application to a series of complexes of the dopamine D3 receptor. As we have illustrated, it is very challenging to model with high confidence the thermodynamics of enclosed water molecules in protein binding sites. While more research is needed to improve and automate model parameterization and model accuracy, this study confirms that it is both useful and viable to include enclosed hydration effects in binding free energy calculations with implicit solvation as an alternative to explicit modeling, which is more affected by slow equilibration. [66–68]

The experimental dissociation constants and the computational modeling work have provided valuable insights for the design of stronger and specific ligands of the dopamine D3 receptor. This study emphasizes the benefits of interdisciplinary approaches by tackling difficult rational drug design problems from different experimental, synthetic and modeling sides.

## Supporting information

S1 File Chemistry and synthesis. (PDF)
