# Supplementary material for: Inclusion of enclosed hydration effects in the binding free energy estimation of dopamine D3 receptor complexes
Source: PLoS One. 2019 Sep 30;14(9):e0222902. doi: 10.1371/journal.pone.0222902 (PMC6768453; doi:10.1371/journal.pone.0222902)
Supplement: S1 File — (PDF) [file pone.0222902.s001.pdf]

Supplementary Information:

Inclusion of Enclosed Hydration Effects in the Binding Free Energy Estimation of Dopamine D3 Receptor Complexes

Rajat Kumar Pal[1,5], Steve Ramsey[2,5], Satishkumar Gadhiya[4,6], Pierpaolo Cordone[4,5], Lauren Wickstrom[3], Wayne W. Harding[4,5,6], Tom Kurtzman[2,5,6], Emilio Gallicchio[1,5,61]

S1 Chemistry and Synthesis

4-(benzyloxy)-3-hydroxybenzaldehyde (5)

To a stirring solution of 3,4-dihydroxybenzaldehyde, 4 (2.5 g, 18.1 mmol) in anhydrous Acetonitrile (50 mL), was added K_2CO_3 (2.5 g, 18.1 mmol) followed by benzyl bromide (3.2 mL, 27.15 mmol) slowly, at room temperature, under an inert atmosphere. Resulting reaction mass was heated to reflux temperature and stirring was continued for 2 h. The reaction solvent was removed by evaporation under reduced pressure and to the resulted residue was added cold 10% NaOH solution and stirred for 10 min. and added the ethyl acetate (2 x 50 mL). Resulted biphasic mixture was separated and aqueous layer was acidified using 4N HCl and extracted with DCM (3 x 30 mL), combined organic layer was washed with brine solution, water, dried on Na_2SO_4, and concentrated under reduced pressure to obtain the residue, which was purified by crystallization using ethylacetate to afford, 5 (2.7 g, 65%); ^{1}H NMR (400 MHz, $CDCl_3$) δ 9.83 (s, 1H), 7.46-7.38 (m, 7H), 7.03 (d, J = 8.2 Hz, 1H), 5.81 (s, 1H), 5.20 (s, 2H); ^{13}C NMR (400 MHz, $CDCl_3$) δ 190.9, 150.9, 146.3, 135.2,

[1]Corresponding author: egallicchio@brooklyn.cuny.edu. [1]Department of Chemistry, Brooklyn College, 2900 Bedford Avenue, Brooklyn, New York 11210. [2]Department of Chemistry, Lehman College, The City University of New York, 250 Bedford Park Blvd. West, Bronx, New York, NY, 10468. [3]Borough of Manhattan Community College, Department of Science, The City University of New York, 199 Chambers Street, New York, NY, 10007. [4]Department of Chemistry, Hunter College, The City University of New York, NY. [5]Ph.D. Program in Biochemistry, The Graduate Center of the City University of New York, New York, NY 10016. [6]Ph.D. Program in Chemistry, The Graduate Center of the City University of New York, New York, NY 10016.

130.8, 128.9 X 2, 128.8, 127.9 X 2, 124.3, 114.4, 111.5, 71.2; HRMS (ESI) m/z calcd for $C_{14}H_{12}O_3$ ($[M + H]^+$), 229.0859, found 229.0863.

2-(benzyloxy)-5-(2-nitrovinyl)phenoxy)(tert-butyl)diphenylsilane (6)

Compound 5 (2.7 g, 11.8 mmol) was dissolved in anhydrous DCM (50 mL). Imidazole (1.6 g, 23.6 mmol) and DMAP (0.1 g, 0.12 mmol) were added in to reaction mixture and allowed it to stir at 0 $^\circ$ C for 10 minute. To the reaction mixture, TBDPSCl (4.6 mL 17.7 mmol) was added and the reaction was allowed to stir overnight at rt. Reaction mixture was acidified with 2N HCl and extracted with (2 x 40 mL) DCM. Combined organic layer was washed with brine solution, water, dried on Na_2SO_4 and concentrated under reduced pressure to obtain the crude residue, which was used in the next step without further purification. Residues were dissolved in 50 mL acetic acid, NH_4OAc (1.0 , 12.98 mmol) and CH_3NO_2 (2.5, 47.2 mmol) were added in it and allowed it to reflux for 8 hr. Solvent was evaporated and the residues were basified using 10% NaOH and extracted with DCM (2 x 50 mL). Combined organic layer was dried over Na_2SO_4, and concentrated under reduced pressure to obtain a crude residue, which was purified by column chromatography on silica gel using 10:90 EtOAC: Hexanes as eluent to afford compound 6 (5.1 g, 85% over two steps); ^1H NMR (400 MHz, $CDCl_3$) δ 7.73-7.63 (m, 5H), 7.46-7.27 (m, 11H), 7.05-7.00 (m, 2H), 6.86-6.77 (m, 2H), 4.97 (s, 2H), 1.10 (s, 9H); 13C NMR (400 MHz, $CDCl_3$) δ 153.5, 145.8, 139.1, 136.1 X 2, 135.4 X 2 , 135.3 X 2, 135.0, 134.8, 132.8, 130.1, 129.7, 128.5 X 3, 128.0 X 2, 127.8 X 4, 127.4 X 2, 124.8, 122.7, 119.5, 113.5, 70.5, 26.6; HRMS (ESI) m/z calcd for $C_{31}H_{31}NO_4Si$ ($[M+H]^+$), 510.2101, found 510.2098.

2-(4-(benzyloxy)-3-((tert-butyldiphenylsilyl)oxy)phenyl)ethan-1-amine (7)

In a dry round bottom flask, $LiBH_4$ (1.8 g, 82.4 mmol), was suspended in THF (30 mL) and TMSCl (5.3 mL, 41.2 mmol) was added slowly. Solution of compound 6 (5.1 g, 10.3 mmol) in THF (30 mL) was added slowly in to reaction mixture and allowed to reflux overnight.

Reaction was allowed to cool at rt and excess $LiBH_4$ was quenched with MeOH. 10% NaOH (20 mL) was added to the reaction and it was filtered through celite. Solvent was evaporated and the crude mixture was extracted with DCM (2 x 50 mL). Combined organic layer was dried over Na_2SO_4, and concentrated under reduced pressure to obtain compound 7; ^{1}H NMR (400 MHz, $CDCl_3$) δ 7.72-7.68 (m, 5H), 7.41-7.27 (m, 10H), 6.74 (d, J = 6.6 Hz, 1H) 6.62 (dd, J = 1.4, 6.5 Hz, 1H), 6.4 (d, J = 1.5 Hz, 1H), 4.88 (s, 2H), 2.72 (t, J = 5.8 Hz, 2H), 2.59 (t, J = 5.8 Hz, 2H), 1.08 (s, 9H); 13C NMR (400 MHz, $CDCl_3$) δ 147.5, 144.4, 136.1, 134.4 X 2 , 135.3 X 2, 133.7, 132.2, 129.1, 128.7 X 3, 128.6, 127.2 X 4, 126.8 X 2, 126.7, 126.4 X 2, 120.7, 119.7, 113.2 X 2, 69.6, 40.9, 34.5, 25.5; HRMS (ESI) m/z calcd for $C_{31}H_{35}NO_2Si$ ($[M+H]^+$), 482.2515, found 482.2509.

2-(4-(benzyloxy)-2-(hydroxymethyl)-3-methoxyphenyl)-N-(4-(benzyloxy)-3-((tert-butyldiphenylsilyl)oxy)phenethyl) acetamide (9)

Compound 8 (1.5 g, 5.3 mmol) and primary amine 7 (2.5g, 5.3 mmol) were refluxed in to ethanol (20 mL) for 16 h. Solvent was evaporated and the residues were purified by column chromatography (2 % MeOH/DCM) to yield 10 (3.2g, 78%). ^{1}H NMR ($CDCl_3$, 600 MHz) δ 7.69 (dd, J = 8.1, 1.3 Hz, 4H), 7.43-7.36 (m, 6H), 7.33-7.28 (m, 10H), 6.82 (d, J = 8.4 Hz, 1H), 6.72 (d, J = 8.4 Hz, 1H), 6.70 (d, J = 8.1 Hz, 1H), 6.42-6.38 (m, 2H), 5.56 (s, 1H), 5.10 (s, 2H), 4.93 (s, 2H), 4.64 (d, J = 6.2 Hz, 2H), 3.93 (s, 3H), 3.55 (t, J = 6.2 Hz, 1H), 3.35 (s, 2H), 3.11 (q, J = 6.4 Hz, 2H), 2.41 (t, J = 6.6 Hz, 2H), 1.09 (s, 9H); 13C NMR ($CDCl_3$, 150 MHz) δ 171.4, 151.1, 148.7, 145.5, 137.3, 137.0, 135.5 X 3, 134.2, 133.4, 131.4, 129.8 X 3, 128.6 X 3, 128.3 X 3, 128.0 X 3, 127.7 X 2, 127.6 X 2, 127.4 X 2, 127.2 X 2, 125.6, 121.8, 120.6, 114.3, 114.1, 70.8 X 2, 61.8, 56.6, 40.7, 40.6, 34.3, 26.6 X 3, 19.7; (ESI) m/z calcd. for $C_{48}H_{51}NO_6Si$ ($[M+Na]^+$), 788.3378, found 788.3416.

3-(benzyloxy)-6-(2-((4-(benzyloxy)-3-((tert-butyldiphenylsilyl)oxy)phenethyl)amino)-2-oxoethyl)-2-methoxybenzyl acetate (10)

To the solution of compound 10 (3.1 g, 4.1 mmol) in DCM (20 mL) was added pyridine (0.6 mL, 8.2 mmol) and DMAP (10 mg, 0.08 mmol). The mixture was cooled to 0 $^\circ$ C and acetic anhydride (0.8 mL, 8.2 mmol) was added dropwise. The solution was allowed to stir for 1.5 h at room temperature, the reaction mixture was diluted with DCM (20 mL), washed with 1 N HCl and dried over sodium sulfate and concentrated. The residue was purified by flash chromatography (1.5% MeOH/DCM) to afford 10 (3.1 g, 92%). ^{1}H NMR ($CDCl_3$, 500 MHz) δ 7.66 (dd, J = 8.1, 1.3 Hz, 4H), 7.41-7.36 (m, 6H), 7.33-7.27 (m, 10H), 6.88 (d, J = 8.4 Hz, 1H), 6.78 (d, J = 8.4 Hz, 1H), 6.68 (d, J = 8.4 Hz, 1H), 6.40-6.38 (m, 2H), 5.14 (s, 2H), 5.08 (s, 2H), 4.88 (s, 2H), 3.88 (s, 3H), 3.45 (s, 2H), 3.10 (q, J = 6.7 Hz, 2H), 2.40 (t, J = 6.8 Hz, 2H), 1.97 (s, 3H), 1.07 (s, 9H);); ^{13}C NMR ($CDCl_3$, 125 MHz) δ 170.8, 170.5, 151.1, 149.2, 148.3, 145.4, 137.2, 136.6, 135.4 X 3, 133.3, 131.2, 129.7 X 3, 128.6 X 3, 128.3 X 3, 128.0 X 3, 127.6 X 4, 127.4 X 2, 127.2 X 2, 126.2, 121.6, 120.5, 114.8, 114.1, 70.7 X 2, 61.4, 58.4, 40.5, 40.4, 34.5, 26.6 X 3, 20.9, 19.7; (ESI) m/z calcd. for $C_{50}H_{53}NO_7Si$ ($[M+Na]^+$), 830.3489, found 830.3491.

(S)-3-(benzyloxy)-6-((7-(benzyloxy)-6-hydroxy-1,2,3,4-tetrahydroisoquinolin-1-yl)methyl)-2-methoxybenzyl acetate (11)

To the solution of 10 (3.0 g, 3.7mmol) in dry acetonitrile (20 mL) was added $POCl_3$ (2.1 mL, 22.3 mmol) and the mixture was refluxed for 1 h. The reaction mixture was cooled to room temperature and the solvent was evaporated. The residue was dissolved in DCM (30 mL) and washed with saturated $NaHCO_3$ (15 mL). The organic layer was dried over sodium sulfate and evaporated to dryness. The yellow solid residue was dissolved in DMF (15 mL) and RuCl[(R,R)-TsDPEN(P-cymene)] (20 mg, 0.03 mmol) was added under nitrogen. The

solution was purged Nitrogen gas for 15 minutes. The mixture of formic acid (0.8 mL, 20.7 mmol) and triethyl amine (0.3 mL 2.21 mmol) was added in to the reaction mixture and allowed it to stir overnight at rt. The reaction mixture was adjusted pH to 8 with saturated $NaHCO_3$ and extracted with ethyl acetate (3 X 35 mL). The combined organic layer was washed with brine, dried over Na_2SO_4 and concentrated. The crude product was purified by column chromatography on silica gel using 3:97 to 5:95 MeOH:DCM as eluent to afford compound, 11 (1.4 g, 67% from two steps).

(S)-2,10-bis(benzyloxy)-9-methoxy-5,8,13,13a-tetrahydro-6H-isoquinolino[3,2-a]isoquinolin-3-ol (12)

Compound 11 (1.4 g, 2.5 mmol) was dissolved in ethanol (20 mL) and added 10% NaOH (10 mL) to the solution then the mixture was refluxed for 1 h. The mixture was cooled and ethanol was evaporated. The residue was extracted with ehyl acetate (2 X 30 mL) and the combined organic layer was washed dried over Na_2SO_4 and evaporated to dryness. The residues were dissolved in dry DCM (15 mL) and the solution was cooled 0 ° C. Thionyl chloride (0.9 mL, 12.5 mmol) was added dropwise to the reaction mixture and allowed it to stir for 1 hr. Reaction solution was cooled to 0 ° C and saturated $NaHCO_3$ (30 mL) was added to it and allowed it stir for 1 h. Reaction mixture was extracted with DCM (2 X 50 mL), combined organic layer was dried over sodium sulfate and evaporated to dryness. The crude product was purified by column chromatography on silica gel using 1:99 MeOH:DCM as eluent to afford compound, 12 (1.1 g, 88% over two steps).^{1}H NMR ($CDCl_3$, 500 MHz) δ 7.45-7.37 (m, 10H), 6.83 (s, 2H), 6.78 (s, 1H), 6.71 (s, 1H), 5.58 (s, 1H), 5.14-5.06 (m, 4H), 4.25 (d, J = 15.8 Hz, 1H), 3.90 (s, 3H), 3.55-3.52 (m, 2H), 3.20-3.08 (m, 3H), 2.80 (dd, J = 11.5, 15.4 Hz, 1H), 2.67-2.60 (m, 2H); ^{13}C NMR ($CDCl_3$, 120 MHz) δ 149.3, 145.6, 144.3, 144.2, 137.2, 136.4, 129.2, 128.9, 128.7 X 2, 128.5 X 2, 128.4, 128.2, 128.0, 127.9, 127.8 X 2, 127.3 X 2, 123.7, 114.4, 113.1, 109.4, 71.4, 70.9, 60.3, 59.3, 54.0, 51.5, 36.5, 28.9; (ESI) m/z calcd. for $C_{32}H_{31}NO_4$ ($[M+H]^+$), 494.2326, found 494.2330.

General synthetic procedure for the compounds 1a-1f as demonstrated for 1a (S)-3-ethoxy-9-methoxy-5,8,13,13a-tetrahydro-6H-isoquinolino[3,2-a]isoquinoline-2,10-diol (1a)

Compound, 12 (1.0 eq) was dissolved in anhydrous DMF and added K_2CO_3 (2.0 eq) followed by ethyl bromide (1.2 eq). Resulted reaction mass was allowed to stir at rt for 5 h. The reaction mass was quenched with cold water and extracted with ethyl acetate (3 x 50 mL). Combined organic layer was washed with brine solution, dried over Na_2SO_4 and concentrated to obtain a residue, which was used in the next step without further purification. Residues were dissolved in ethanol (10 mL) and refluxed with conc. HCl (4 mL) for 3 hr. The reaction mixture was concentrated in vacuo and residue was basified using aqueous ammonia solution and extracted with ethyl acetate (2 X 20 mL). Combined organic layer was washed with brine solution, dried over Na_2SO_4 and concentrated under reduced pressure to obtain a residue, which was purified by column chromatography on silica gel using 2:98 MeOH:DCM as eluent to afford compound 1a. Yield 71 %, white solid, Mp 104 ^{0}C; ^{1}H NMR ($CDCl_3$, 500 MHz) δ 6.84-6.79 (m, 3H), 6.59 (s, 1H), 4.20 (d, J = 15.4 Hz, 1H), 4.10 (q, J = 7.0 Hz, 2H), 3.81 (s, 3H), 3.58-3.53 (m, 2H), 3.27-3.09 (m, 3H), 2.80 (dd, J = 11.4, 15.9 Hz, 1H), 2.67-2.62 (m, 2H), 1.45 (t, J = 7.0, 3H); ^{13}C NMR ($CDCl_3$, 125 MHz) δ 146.4, 144.3, 144.0, 143.1, 130.3, 127.9, 127.3, 125.8, 125.0, 114.0, 111.4, 111.2, 64.4, 60.7, 59.3, 53.9, 51.7, 36.2, 29.1, 14.0; (ESI) m/z calcd. for $C_{20}H_{23}NO_4$ ($[M+H]^+$), 342.1700, found 342.1697.

(S)-9-methoxy-3-propoxy-5,8,13,13a-tetrahydro-6H-isoquinolino[3,2-a] isoquinoline-2,10-diol (1b)

Yield 75%, dark brown solid, Mp 82 ^{0}C; ^{1}H NMR ($CDCl_3$, 500 MHz) δ 6.85-6.81 (m, 3H), 6.59 (s, 1H), 4.28 (d, J = 15.4 Hz, 1H), 3.99 (t, J = 6.6 Hz, 2H), 3.82 (s, 3H), 3.65-3.58 (m, 2H), 3.28-3.24 (m, 2H), 3.15 (td, J = 5.6, 16.7 Hz, 1H), 2.84 (dd, J = 11.8, 16.0 Hz, 1H), 2.70-2.66 (m, 2H), 1.84 (sext, J = 7.1 Hz, 2H), 1.05 (t, J = 7.5, 3H); ^{13}C NMR ($CDCl_3$, 125 MHz) δ 146.5, 144.5, 144.1, 143.2, 130.3, 127.9, 127.3, 125.8, 125.0, 114.2, 111.5, 111.2, 70.4,

60.7, 59.4, 53.9, 51.7, 36.1, 29.1, 22.6, 10.5; (ESI) m/z calcd. for $C_{21}H_{25}NO_4$ ($[M+H]^+$), 356.1856, found 356.1849.

(S)-3-butoxy-9-methoxy-5,8,13,13a-tetrahydro-6H-isoquinolino[3,2-a] isoquinoline-2,10-diol (1c)

Yield 76%, yellowish powder, Mp 85 ^{0}C; ^{1}H NMR ($CDCl_3$, 500 MHz) δ 6.85-6.81 (m, 3H), 6.59 (s, 1H), 4.28 (d, J = 15.4 Hz, 1H), 3.99 (t, J = 6.6 Hz, 2H), 3.82 (s, 3H), 3.65-3.58 (m, 2H), 3.28-3.24 (m, 2H), 3.15 (td, J = 5.6, 16.7 Hz, 1H), 2.84 (dd, J = 11.8, 16.0 Hz, 1H), 2.70-2.66 (m, 2H), 1.84 (sext, J = 7.1 Hz, 2H), 1.05 (t, J = 7.5, 3H); ^{13}C NMR (CDCl3, 125 MHz) δ 146.5, 144.5, 144.0, 143.1, 130.2, 127.8, 127.3, 125.8, 125.0, 114.2, 111.4, 111.1, 68.6, 60.7, 59.4, 53.9, 51.7, 36.1, 31.3, 29.1, 19.2, 13.8; (ESI) m/z calcd. for $C_{22}H_{27}O_4$ ($[M+H]^+$), 370.2013, found 370.2006.

(S)-9-methoxy-3-(pentyloxy)-5,8,13,13a-tetrahydro-6H-isoquinolino[3,2-a] isoquinoline-2,10-diol (1d)

Yield 73%, brown solid, Mp 73 ^{0}C; ^{1}H NMR ($CDCl_3$, 500 MHz) δ 6.83-6.79 (m, 3H), 6.59 (s, 1H), 5.56 (s, 1H), 4.20(d, J = 15.3 Hz, 1H), 4.02 (t, J = 6.6 Hz, 2H), 3.81 (s, 3H), 3.58-3.55 (m, 2H), 3.27-3.10 (m, 3H), 2.80 (dd, J = 11.6, 15.8 Hz, 1H), 2.67-2.62 (m, 2H), 1.82 (pent, J = 6.6 Hz, 2H), 1.48-1.36 (m, 4H), 0.94 (t, J = 7.2, 3H); ^{13}C NMR ($CDCl_3$, 125 MHz) δ 146.4, 144.5, 144.1, 143.1, 130.2, 127.8, 127.3, 125.8, 125.0, 114.1, 111.4, 111.1, 68.9, 60.7, 59.4, 53.9, 51.7, 36.1, 29.0, 28.9, 28.2, 22.4, 14.0 ; (ESI) m/z calcd. for $C_{23}H_{29}NO_4$ ($[M+H]^+$), 384.2169, found 384.2165.

(S)-3-(hexyloxy)-9-methoxy-5,8,13,13a-tetrahydro-6H-isoquinolino[3,2-a] isoquinoline-2,10-diol (1e)

Yield 70%, dark brown powder, Mp 61 ^{0}C; ^{1}H NMR ($CDCl_3$, 500 MHz) δ 6.83-6.79 (m, 3H), 6.59 (s, 1H), 4.22 (d, J = 15.3 Hz, 1H), 4.02 (t, J = 6.6 Hz, 2H), 3.81 (s, 3H), 3.58(d, J

= 14.7 Hz, 2H), 3.28-3.12 (m, 3H), 2.84-2.79 (m, 1H), 2.69-2.64 (m, 2H), 1.81 (pent, J = 6.7 Hz, 2H), 1.49-1.43 (m, 2H), 1.34 (sext, J = 3.7, 4H), 0.91 (t, J = 7.0, 3H); ^{13}C NMR ($CDCl_3$, 125 MHz) δ 146.4, 144.5, 144.0, 143.1, 130.2, 127.9, 127.3, 125.8, 125.0, 114.1, 111.4, 111.1, 68.9, 60.7, 59.4, 53.9, 51.7, 36.2, 31.5, 29.2, 29.1, 25.7, 22.6, 14.0; (ESI) m/z calcd. for $C_{24}H_{31}NO_4$ ([M+Na]$^+$), 398.2326, found 398.2319.

(S)-3-(2-fluoroethoxy)-9-methoxy-5,8,13,13a-tetrahydro-6H-isoquinolino[3,2-a] isoquinoline-2,10-diol (1f)

Yield 87%, yellow powder, Mp 95 ^{0}C; ^{1}H NMR ($CDCl_3$, 500 MHz) δ 6.85-6.79 (m, 3H), 6.61 (s, 1H), 4.82 (t, J = 3.9 Hz, 1H), 4.73 (t, J = 3.9 Hz, 1H), 4.32-4.19 (m, 3H), 3.81 (s, 3H), 3.58-3.54 (m, 2H), 3.27-3.10 (m, 3H), 2.80 (dd, J = 11.5, 15.7 Hz, 1H), 2.68-2.62 (m, 2H); ^{13}C NMR ($CDCl_3$, 125 MHz) δ 146.4, 144.3, 143.8, 143.1, 131.5, 127.8, 127.2, 126.0, 125.0, 114.2, 112.4, 111.9, 82.4, 81.0, 68.5, 68.3, 60.7, 59.3, 53.8, 51.6, 36.0, 29.0; (ESI) m/z calcd. for C20H22FNO4 $C_{20}H_{22}FNO_4$ ([M+Na]$^+$), 360.1606, found 360.1599.

(±)-Stepholidine (2.1)

Lithium aluminum hydride (40 mg, 1.1 mmol) was stirred in THF at 0° C for 10 min. Compound 3.11 (0.3 g, 0.7 mmol) in THF was added to the reaction mixture at 0° C and it was allowed to reflux for 2 h. The reaction mixture was allowed to cool to rt and excess of lithium aluminum hydride was quenched with water. THF was evaporated and crude mixture was extracted with dichloromethane. The organic layer was dried over sodium sulfate, filtered and evaporated to dryness. The crude product was refluxed in mixture of methanol (5 mL) and concentrated hydrochloric acid (5 mL) for 3 h. The reaction mixture was basified by ammonia solution and extracted with dichloromethane (20 mL $\times$ 2). The combined organic layer was dried over sodium sulfate and concentrated to give the crude product, which was purified by flash column chromatography on silica gel (2%-6% methanol/dichloromethane) to give compound 2.1 (0.29 g, 81% over two steps): Yellowish white crystals, mp 157° C; ^{1}H

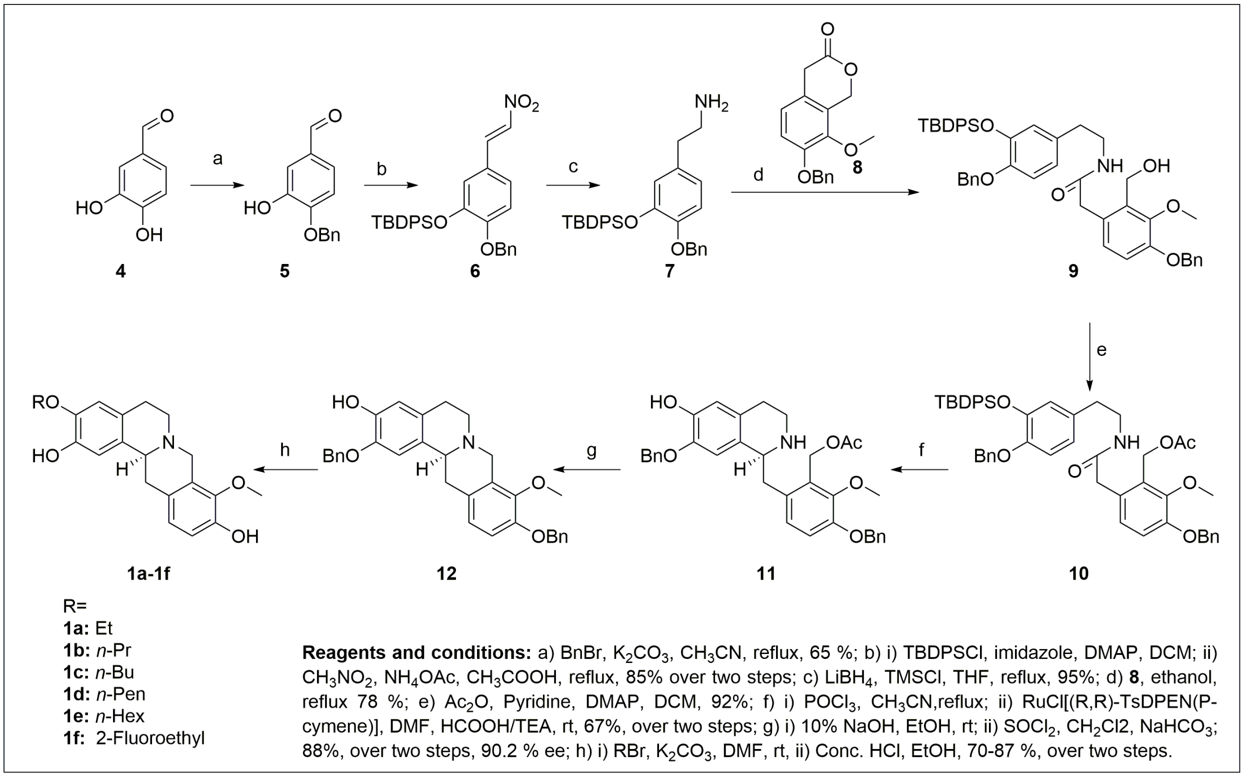

Figure A: Synthesis of C3 analogues of (-)-stepholidine

NMR ($CDCl_3$, 400MHz) δ 6.83-6.78 (m, 3H), 6.60 (s, 1H), 4.20 (d, J = 15.8 Hz, 1H), 3.87 (s, 3H), 3.81 (s, 3H), 3.58-3.53 (m, 2H), 3.27-3.10 (m, 3H), 2.80 (dd, J = 15.9, 11.4 Hz, 1H), 2.70-2.62 (m, 2H); ^{13}C NMR ($CDCl_3$, 400MHz) δ 146.4, 145.1, 144.0, 143.1, 130.4, 127.8, 127.3, 125.9, 125.0, 114.1, 111.3, 110.6, 60.7, 59.3, 55.9, 58.9, 51.7, 36.1, 29.1; HRMS (ESI) m/z calcd. for C19H21NO4 $C_{19}H_{21}NO_4$ ($[M+H]^+$), 328.1551, found 328.1543.

Table A: Experimental and calculated energetic quantities with uncertainties. Experimental and calculated binding free energies, average binding energies and reorganization free energies of the (-)-stepholidine C3 analogues with and without enclosed hydration corrections.

Ligands	Structure	$\Delta G^{\circ a}_{\text{exp}}$	Without enclosed hydration model			With enclosed hydration model		
			$\Delta G^{\circ a}_{\text{calc}}$	ΔE^{a}_{b}	$\Delta G^{\circ a}_{\text{reorg}}$	$\Delta G^{\circ a}_{\text{calc}}$	ΔE^{a}_{b}	$\Delta G^{\circ a}_{\text{reorg}}$
1a	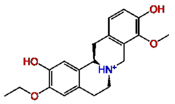	-10.1	-2.2 ± 0.07	-36.9 ± 0.22	34.7 ± 0.24	-8.8 ± 0.07	-42.5 ± 0.21	33.7 ± 0.23
1b	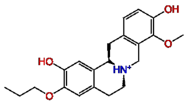	-10.0	-2.3 ± 0.07	-38.0 ± 0.22	35.7 ± 0.23	-10.4 ± 0.07	-44.7 ± 0.19	34.3 ± 0.20
1c	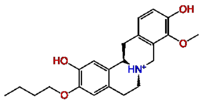	-10.0	-1.8 ± 0.08	-40.3 ± 0.23	38.5 ± 0.24	-11.5 ± 0.08	-48.1 ± 0.18	36.6 ± 0.19
1d	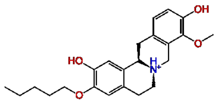	-10.2	-0.3 ± 0.09	-43.7 ± 0.26	43.4 ± 0.29	-10.6 ± 0.08	-55.6 ± 0.22	45.0 ± 0.24
1e	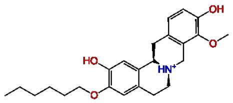	-10.4	-3.9 ± 0.11	-39.6 ± 0.21	35.7 ± 0.23	-12.5 ± 0.08	-55.2 ± 0.22	42.7 ± 0.23
1f	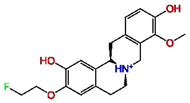	-9.64	-3.1 ± 0.11	-32.7 ± 0.27	29.6 ± 0.29	-8.9 ± 0.07	-43.2 ± 0.19	34.3 ± 0.20

[a] All values in kcal/mol.
